# Defective RNA of a Novel Mycovirus with High Transmissibility Detrimental to Biocontrol Properties of *Trichoderma* spp.

**DOI:** 10.3390/microorganisms7110507

**Published:** 2019-10-29

**Authors:** Jiaqi You, Kang Zhou, Xiaolin Liu, Mingde Wu, Long Yang, Jing Zhang, Weidong Chen, Guoqing Li

**Affiliations:** 1 State Key Laboratory of Agricultural Microbiology, Huazhong Agricultural University, Wuhan 430070, Chinaxiaolinliu1996@163.com (X.L.); yanglong@mail.hzau.edu.cn (L.Y.); zhangjing1007@mail.hzau.edu.cn (J.Z.); guoqingli@mail.hzau.edu.cn (G.L.); 2 The Key Laboratory of Plant Pathology of Hubei Province, Huazhong Agricultural University, Wuhan 430070, China; 3 Horticultural Research Institute, Shanghai Academy of Agricultural Sciences, Shanghai 201106, China; 4 U.S. Department of Agriculture, Agricultural Research Service, Washington State University, Pullman, WA 99164, USA; w-chen@wsu.edu

**Keywords:** biological control, *Trichoderma* spp., hypovirus, *Botrytis cinerea*

## Abstract

*Trichoderma* species are a group of fungi which is widely distributed in major terrestrial ecosystems; they are also commonly used as biocontrol agents for many plant diseases. A virus, namely Trichoderma harzianum hypovirus 1 (ThHV1), was identified in *T. harzianum* isolate T-70, and also infected isolate T-70D, together with its defective RNA (ThHV1-S). The ThHV1 genome possessed two Open Reading Frames (ORFs), namely ORF1 and ORF2. The start codon of ORF2 overlapped with the stop codon of ORF1 in a 43 nt long region. The polypeptide encoded by ORF2 of ThHV1 shared sequence similarities with those of betahypoviruses, indicating that ThHV1 is a novel member of *Hypoviridea*. Isolate T-70D, carrying both ThHV1 and ThHV1-S, showed abnormal biological properties, notably a decreased mycoparasitism ability when compared with isolate T-70. Both ThHV1 and ThHV1-S could be vertically transmitted to conidia and horizontally transmitted to *T. harzianum* isolate T-68 and *T. koningiopsis* T-51. The derivative strains carrying both ThHV1 and ThHV1-S showed decreased mycoparasitism ability, whereas strains carrying ThHV1 alone were normal, indicating that ThHV1-S is closely associated with the decreased mycoparasitism ability of *T. harzianum* isolate T-70D. ThHV1 was widely detected in isolates of *T. harzianum*, *T. koningiopsis* and *T. atroviride* originating from soil of China. Therefore, viruses in fungal biocontrol agents may also be a factor associated with the stability of their application.

## 1. Introduction

*Trichoderma* species are ubiquitous in soil, plant debris, and the fungal biomass in the vast majority of terrestrial ecosystems including forests, grasslands, and agroecosystems [1]. They possess various abilities in suppressing plant diseases, including the production of a wide range of antibiotic substances, parasitism on other fungi, competition with other microorganisms, the promotion of plant growth, and the induction of plant resistance [2,3]. Consequently, *Trichoderma* spp. has been widely used for the biological control of plant diseases, especially soil-borne diseases [3,4,5], accounting for more than 60% of the registered fungus-based biofungicides [6]. Besides agricultural use, they also have been used in industry, e.g, enzyme production, paper and pulp treatment, bioremediation, etc. [7]. Nevertheless, the biocontrol of plant disease, including the application of *Trichoderma* spp., was generally believed to be unstable under field conditions [8], thus limiting its use. Many factors were associated with the efficacy of biological control agents (BCAs), such as environmental conditions (temperature and relative humidity), stability of BCA, and timing for application of BCA, etc. [9]. In addition, biotic factors, like the microbial communities (microbiota) in the phyllosphere and rhizosphere, were also supposed to have a direct or indirect influence on the biocontrol efficacy, although experimental evidence is limited [10].

Viruses that inhabit and replicate in filamentous fungi, yeasts, and oomycetes are designated as mycoviruses or fungal viruses [11]. Although the first mycovirus was only discovered about 50 years ago [12], mycoviruses are widely observed in all major fungal groups; it has been proposed that 30–80% of fungal species may be infected with mycoviruses [11]. Most identified mycoviruses have the genomes of linear double-stranded RNA (dsRNA) or + single-stranded RNA (+ssRNA); few members possess linear -ssRNA or circular ssDNA [12]. Although many virus-infected fungi lack discernable phenotypic changes [13], the infection of some mycoviruses showed harmful or beneficial effects on their hosts. For example, some mycoviruses are able to reduce disease in plants caused by host fungi, termed as hypovirulence, or cause disease on cultivated mushroom [14]. Thus, some hypovirulence-inducing mycoviruses have been used as BCA for plant fungal disease, notably, Cryphonectria hypovirus 1 (CHV1), which has been successfully used for the control of chestnut blight in Europe [15]. On the other hand, some mycoviruses could also offer benefits to their hosts. In some cases, mycoviral infection can enhance the competitive ability of their hosts by producing killer proteins, like the killer viruses in *Saccharomyces cerevisiae* and *Ustilago maydis* [14], or be able to increase the virulence (hypervirulence) of the host fungi [16]. Furthermore, one mycovirus, namely Curvularia thermal tolerance virus (CThTV), also participates in a complex three-way mutualistic symbiosis. The panic grass *Dichanthelium lanuginosum* requires a fungal endophyte, *Curvularia protuberata*, carrying the mycrovirus CThTV, to survive at soil temperatures >50 °C in Yellowstone National Park, USA [17]. Although previous studies have well demonstrated that mycoviruses may have different effects on different fungal groups (phytopathogenic fungi, mushrooms, and endophytes), few mycoviruses were reported in fungus-based BCAs, and their effects on biocontrol activity remain unknown, with the exception of a recent report of hypervirulence-related mycoviruses in the entomopathogenic fungus *Beauveria bassiana* [18]. As mycoviruses are widely distributed in different fungal groups and possess various ecological functions, viruses may also exist in the population of fungus-based BCAs, and positively or negatively affect the biocontrol efficacy.

Hypoviruses are a group of +ssRNA mycovirus with genome sizes ranging from 9 to 13 kb, excluding the poly (A) tail. The genomes of hypoviruses generally encompass two minimally overlapping ORFs or one large ORF [12,19]. Defective interfering RNAs (DI-RNA) are subviral RNAs generated during the multiplication of RNA viruses by error-prone viral replicases, as they have lost the capacity for independent replication, and usually attenuate the symptoms caused by the helper virus through impairing the replication of the helper viral RNAs [20]. Moreover, defective RNAs that do not interfere with the replication of intact cognate viral RNAs are simply termed as D-RNAs [21]. DI-RNAs are frequently observed in hypoviruses, especially for Cryphonectria hypoviruses, and the generation of those DI RNAs have been determined to be associated with two host dicer genes [22].

In this study, two dsRNAs of approximately 11 and 10 kb in length were detected in the *T. harzianum* isolate T-70D, while only the 11-kb dsRNA was detected in the original isolate T-70 of *T. harzianum*. Genomic analysis showed that the 11-kb dsRNA corresponded to a novel hypovirus, namely Trichoderma harzianum hypovirus 1 (ThHV1), while the 10-kb dsRNA was the defective RNA of ThHV1, namely ThHV1-S. Compared with isolate T-70, the mycoparasitic ability of isolate T-70D decreased dramatically. Therefore, this study aimed (i) to obtain and analyze the full-length cDNA sequences of ThHV1 and ThHV1-S, (ii) to establish the cause-effect relationship between presence of ThHV1-S and the decreased mycoparasitic ability in isolate T-70D, and (iii) to evaluate the possible effects of ThHV1 on the application of *Trichoderma* spp. for the control of plant disease.

## 2. Materials and Methods

### 2.1. Fungal Isolates/Strains and Mycelial Growth

Ten isolates/strains of *Trichoderma* spp. listed in Table 1 were mainly used in this study. Three field isolates, i.e., T-70 and T-68 of *T. harzianum* and *T. koningiopsis* isolate T-51, were isolated from cropland soil. The field isolates were identified to species level based on the cultural morphology and DNA sequence of the internal transcribed spacer (ITS) region in our previous study [23]. T-70D was obtained from the colony edge of a culture plate of T-70 with an abnormal morphology. Derivative strains originated from isolate T-70D of *T. harzianum* through the single-conidium isolation, or from the corresponding receipt isolates in pair culture with the donor strain T-70D (Table 1). The phytopathogenic fungus *Botrytis cinerea* isolate RoseBC-3 was isolated from a diseased flower of Chinese rose showing gray mold symptoms. All fungal isolates/strains were stored as mycelial plugs in 20% (*v*/*v*) sterilized glycerol solution at −80 °C, and grown on a potato dextrose agar (PDA) plate for subsequent use.

Three mycelial agar plugs of about 5 mm diameter were removed from the colony margin of a two-day-old PDA culture of each *Trichoderma* isolate, and then inoculated on three PDA plates (9 cm diameter). The inoculated plates were incubated at 20 °C, and the diameter of each *Trichoderma* colony was measured on daily basis. The radial growth rate (RGR) of each isolate/strain was determined as described previously [24]. The colony morphology of each *Trichoderma* isolate/strain was observed at 10 days post inoculation. This experiment was repeated at least twice.

### 2.2. Extraction of Nucleic acids

Three-day old (20 °C) mycelial mates of *Trichoderma* isolates/strains on potato dextrose agar (PDA) covered with cellophane film were collected and used for nucleic acid extraction directly, or stored at −80 °C until use. The genomic DNA and total RNA of *Trichoderma* isolates/strains were extracted from the mycelial mates according to the description by Möller [25] and using the TRIzol^®^ reagent (Invitrogen Corp, Carlsbad, CA, USA) [26], respectively. The extracted genomic DNA and total RNA were stored at −20 °C and −80 °C, respectively. The dsRNAs in the mycelia of *Trichoderma* were extracted and purified with CF-11 cellulose (SIGMA-ALDRICH, Inc., Louis, MO, USA) using the method described by Morris and Dodds [27], and stored at −80 °C. The extracted dsRNAs from each *Trichoderma* isolate/strain were separated by agarose gel electrophoresis (0.7%, *w*/*v*). The agarose gel was stained with ethidium bromide solution (1.5 μg/L) and viewed on a UV trans-illuminator. The nature of the dsRNAs was confirmed using the method described previously [24].

### 2.3. cDNA Cloning, Sequencing, and Sequence Analysis of ThHV1 and ThHV1-S

Two dsRNAs of approximately 11.0 and 10.0 kb in length were extracted from the mycelia of isolate T-70D and then gel-purified using the AxyPrep DNA gel extraction kit (Axygen Scientific Inc., Union City, CA, USA). The cDNA library of the 10.0 kb-dsRNA was established through random primer-mediated PCR [28] and sequenced as previously described [29]. The terminal sequences of the two dsRNAs were cloned through ligating the 3′-terminus for each strand of the two dsRNAs with the 5′-terminus of the 110A adaptor (Table S1) using T4 RNA ligase (Promega Corporation, 2800 Woods Hollow Road Madison, WI 53711 USA) at 16 °C for 18 h, and then reverse transcribed using primer RC110A (Table S1) according to our previous methods [26,29]. The cDNA strand was then used as a template for PCR-amplifying the 5′- and 3′-terminal sequence with the primer pairs RC110A/5SP and RC110A/3SP, respectively (Figure S1). Cloning of the 3′- or 5′-terminal sequences of two dsRNAs was repeated at least three times (Figure S1). Three gaps among the cDNA contigs of 10.0 kb-dsRNA were amplified with primer pairs G1F/G1R, G2F/G2R, and G3F/G3R by reverse transcription (RT)-PCR. The cDNA sequence of 11.0 kb-dsRNA without 5′- and 3′-termini were RT-PCR-amplified with primer pairs P5F/P5R, P6F/P6R, P7F/P7R, G1F/G1R, G2F/G2R, and G3F/G3R (Figure S1). All amplicons were separated by agarose gel electrophoresis, cloned into *Escherichia coli* DH5α, and sequenced with the procedures described previously [26]. All partial cDNA sequences were assembled to obtain the full-length cDNA sequence for the two dsRNAs (ThHV1 and ThHV1-S). Open reading frames (ORFs) in full length cDNA sequences of ThHV1 and ThHV1-S were deduced using the ORF Finder program in NCBI (http://www.ncbi.nlm.nih.gov/gorf/) with the standard codon usages. The programs of BLASTn and BLASTp were used for database searches of the full-length cDNA sequences of ThHV1 and ThHV1-S and their deduced polypeptides in the public database at NCBI, respectively. Multiple alignments of the sequences of the RdRps of different viruses were accomplished using the CLUSTAL W program in MEGA 5.0 (www.megasoftware.net) [30]. Three phylogenetic trees based on sequences of RdRp domain, Hel domian, and ORF 2 encoded polypeptide for ThHV1 and other viruses were constructed using the neighbor-joining (NJ) method and tested with a bootstrap using 1000 replicates to ascertain the reliability of a given branch pattern in the NJ trees. The presence of H-type RNA pseudoknots was predicted using the program DotKnot (version 1.3) [31] at the website http://dotknot.csse.uwa.edu.au/.

### 2.4. Northern Hybridization

Northern hybridization was performed to verify the authenticity of the cDNA sequence generated from the two dsRNAs in *T. harzianum* isolate T-70D, and to confirm the 1248 nt genome deletion near the 5′-terminus of ThHV1-S in comparison with ThHV1. The extracted dsRNAs were separated through agarose gel electrophoresis (0.7%, *w*/*v*), and transferred to an Immobilon-Ny+ membrane (Millipore, Bedford, MA, USA) using the method descried previously [29]. Two probes were designed and used in the northern hybridization: Probe 1 (480bp) corresponds to the nucleotide position 1647 to 2126 in the genome of ThHV1 and was absent in ThHV1-S, while Probe 2 (516 bp) corresponds to the nucleotide position 5878 to 6393 in the genome of ThHV1, and was present in both ThHV1 and ThHV1-S genomes. All probes were prelabeled and hybridized with the dsRNAs on Immobilon-Ny+ membrane as described previously [32], and the probe-RNA hybrids’ chemiluminescent signals were detected using a CDP-Star kit (GE Healthcare).

### 2.5. Real-Time Semi-Quantitative PCR

The accumulation levels of ThHV1 and ThHV1-S in isolates T-70 and T-70D of *T. harzianum* were determined using semi-quantitative PCR. Total RNA was extracted from mycelia of each isolate using the E.Z.N.A.^®^ Fungal RNA Kit (Omega Bio-tek, Inc. Norcross, USA). The primer pair TML-F/TML-R (Table S1) was designed for the specific detection of ThHV1 accumulation, as it targets the RT-PCR amplification of the internal-deletion region present only in the genome of ThHV1. The primer pair TMS-F/TMS-R (Table S1) was designed for the specific detection of the ThHV1-S accumulation, as it targets the RT-PCR amplification of the short region in the genome of ThHV1-S. The primer pair TMLS-F/TMLS-R (Table S1) was designed for the detection of the total viral accumulation (ThHV1 + ThHV1-S), as it primed the RT-PCR amplification of a shared region in the genome of ThHV1 and ThHV1-S. Furthermore, the primer pair Tubulin-F/Tubulin-R (Table S1) was used for the specific detection of the β-tubulin gene of *T. harzianum*, which is considered as a constitutive expression gene in fungi, and can be used for justification of the sample-to-sample variation in the amount of RNA [33]. All the PCR amplifications were carried out in a Bio-Rad (Hercules, CA, USA) CFX96TM Real-Time PCR Detection System with SYBR^®^ Premix Ex Taq™ (TaKaRa). The relative accumulation (RA) values of ThHV1 and ThHV1 + ThHV1-S were calculated using the ΔΔCt method following the manufacturer’s instructions.

### 2.6. Vertical and Horizontal Transmission of ThHV1 and ThHV1-S

The vertical transmission of mycoviruses refers to the transmission of mycoviruses through sexual or asexual spores, while the horizontal transmission of mycoviruses refers to the transmission of mycoviruses through hyphal anastomosis [13]. The vertical transmission of ThHV1 was evaluated by detecting ThHV1 and ThHV1-S in single-conidium progenies of T-70D. The conidia of T-70D produced on PDA were washed off with sterile distilled water, and diluted to a concentration of 10^3^ conidia/mL. An aliquot of 100 µL of conidia suspension was spread on a 9-cm PDA plate. The isolation plates were incubated at 20 °C for 24 h. Single-conidium strains were transferred to fresh PDA plates individually for observation of the colony morphology. Ten single conidium-progeny strains were randomly selected for the detection of ThHV1 and ThHV1-S using RT-PCR with the specific primer pairs C1F/C1R and C2F/C2R.

The horizontal transmission of ThHV1 and ThHV1-S in this study was done on PDA plates using the pairing cultural technique, as described previously [29]. Two recipient isolates were used for virus horizontal transmission in this study, i.e., *T. harzianum* isolate T-68, and *T. koningiopsis* isolate T-51. Both isolates have shown great potential as biocontrol agents against *B. cinerea* [23]. Besides isolate T-70D, isolate T-70 was also used for dual culturing with isolate T-51 to test the effect of ThHV1 on *T. koningiopsis* isolate T-51. The pair cultural plate was incubated at 20 °C for 10 days. The mycelial plug from the colony of recipient isolate of T-68 was transferred to a fresh PDA plate to establish the derivative strains, while the derivative strains of isolate T-51 were grown on PDA containing 100 μg/mL hygromycin B, as isolate T-51 was naturally resistant to hygromycin B. Six derivative isolates, i.e., strains 68-1 and 68-3 from T-68, strains 51-12 and 51-13 from T-51 after pairing with T-70D, and strains 51-70-2 and 51-70-4 from T-51 after pairing with T-70, were selected to determine the presence of ThHV1 and ThHV1-S, mycelial growth rate, mycoparasitism, and antifungal activity.

### 2.7. The Mycoparasitism and Antifungal Activity Assay of Trichoderma

A dual-cultural test was conducted to evaluate the ability of *Trichoderma* isolate/strain to parasitize *B. cinerea* as described previously at 20 °C for 10 days [23]. There were three replications for each dual culture treatment. In addition, a semi-quantitative measurement of the relative ability of different *Trichoderma* isolate/strains in parasitizing *B. cinerea* was carried out using the method described previously with some modification [34,35]. From the interface of the mycelia of *Trichoderma* with that of *B. cinerea*, the colony of *B. cinerea* was divided into four approximately 1-cm zones (1, 2, 3, and 4) from the mycelial interface towards the origin of *B. cinerea* colony, as illustrated in Figure 4. Three agar disks from each zone were excised and picked out, plated on PDA, and incubated at 20 °C in light for 7 days to test the emergence of either *Trichoderma* spp. or *B. cinerea* based on the colony morphology [35]. Three types of colonies could be observed, i.e., the *Trichoderma* spp. (Ts) colony alone, the *B. cinerea* (Bc) colony alone, and a complex colony containing both *Trichoderma* spp. and *B. cinerea* (Ts + Bc). Simultaneous growth of *Trichoderma* spp. and *B. cinerea* from the same agar disk suggested that some mycelia of *B. cinerea* in the selected mycelial plugs were still alive in spite of infection by *Trichoderma* spp. The higher frequency of the emergence of *Trichoderma* colonies indicates the higher mycoparasitic ability of the corresponding *Trichoderma* isolate. This experiment was repeated at least twice.

To test the antifungal activity of different *Trichoderma* isolates/strains, the potato dextrose broth (PDB) culture filtrate of each *Trichoderma* isolate/strain was prepared using the method described previously [23]. The mycelial dry weight of *Trichoderma* was measured after drying at 50 °C for 3 days. The inhibition of *B. cinerea* growth on a PDA plate was performed by amending the cultural filtrate in PDA at the volume ratio of 10% (*v*/*v*) before the inoculation of *B. cinerea*. The *B. cinerea* mycelial growth inhibition percentage (GIP) was calculated using the previously-described formula [23].

The PDB cultural filtrate of each *Trichoderma* isolate/strain was also used to test the inhibition of *B. cinerea* development on tomato leaves. Mycelial plugs (5 mm in diameter) from the edge of the *B. cinerea* colony on PDA was inoculated on healthy tomato leaves daubed with 200 μL cultural filtrate after air drying, 3 leaves per treatment, and leaves treated with same volume of PDB were used as a control. The inoculated leaves were incubated at 20 °C for 3 days, and then the lesion diameter on each leaf was measured. This experiment was repeated at least twice.

### 2.8. Suppression of B. cinerea Sporulation on Oilseed Rape Tissues

Healthy leaves of oilseed rape (*Brassica napus* L.) were detached from 60-day-old plants grown in a field. Leaf discs (1 cm diameter) were prepared from the leaves using a cork borer. They were immediately dried at 60 °C for 72 h and then sterilized at 121 °C for 20 min. The sterilized leaf discs were soaked in conidial suspension of *B. cinerea* RoseBc-3 (2 × 106 conidia/mL) and then placed in Petri dishes (9 cm diameter), with 9 leaf discs per dish. As several *Trichoderma* strains formed no conidia, the mycelial plugs (approximately 2 mm in diameter) of each *Trichoderma* strain were inoculated on the leaf discs. The leaf discs inoculated with PDA plugs served as a control. The dishes were placed on the clapboard of a desiccator filled with water at the bottom at 20 °C and 12-h light/12-h dark for four days. The sporulation index of *B. cinerea* on each leaf disc was scored as described previously [23]. The experiment was repeated two more times.

### 2.9. Detection of ThHV1 and ThHV1-S in Trichoderma Population

The total RNAs of 36 *Trichoderma* field isolates were extracted using the TRIzol^®^ reagent (Invitrogen Corp, Carlsbad, CA, USA), and the presence of both ThHV1 and ThHV1-S in these *Trichoderma* isolates of China was determined using RT-PCR with the specific primer pairs C1F/C1R and C2F/C2R (Table S1). The primer pair C1F/C1R was designed to amplify one specific band of 1626 bp and 378 bp in size for ThHV1 and ThHV1-S, respectively, whereas the primer pair C2F/C2R was designed to amplify one specific band of 480 bp in size for ThHV1 and no band for ThHV1-S (Figure S3). Both primer pairs were used for the detection in all 36 *Trichoderma* isolates to confirm the presence of ThHV1 and ThHV1-S.

### 2.10. Statistical Data Analysis

Data of mycelial growth rate, cultural filtrate pH, and the mycelial dry weight of *Trichoderma* isolates/strains, as well as GIP (transfer to arcsine function) and the lesion diameter of *B. cinerea* were analyzed using the SAS program (version 8.1, SAS Institute, Cary, NC, USA). The mean values for different isolates/strains were compared using Duncan’s multiple range test with significance at *p* < 0.05. The mean values between two isolates were compared using Student’s *t*-test, at *p* < 0.05 or *p* < 0.01.

## 3. Results

### 3.1. Nucleotide Sequences and Putative Polypeptides of ThHV1 and ThHV1-S

**Figure 1:**
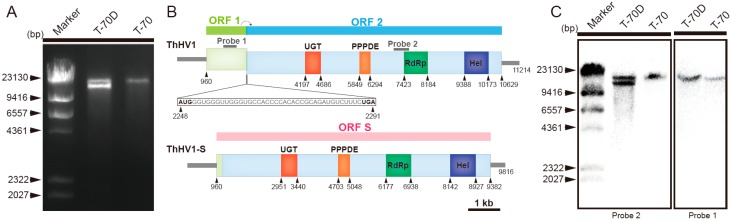
Molecular characterization of Trichoderma harzianum hypovirus 1 (ThHV1) and ThHV1-S. (**A**) Agarose gel electrophoresis of the dsRNAs extracted from the mycelia of *Trichoderma harzianum* isolates T-70 and T-70D. (**B**) Schematic diagrams of the genetic organization of Trichoderma harzianum hypovirus 1 (ThHV1) and ThHV1-S. Note that and two ORFs were overlapped in the 44 nt region of ThHV1, whereas only one ORF was predicted in ThHV1-S. (**C**) Northern blot detection of dsRNAs from the mycelia of isolates T-70 and T-70D using Probe1 and Probe2, respectively.

Two dsRNAs of 11.0 and 10.0 kb in length were detected in the mycelia of isolate T-70D, whereas only the 11.0-kb dsRNA was detected in the mycelia of isolate T-70 (Figure 1A). The full-length cDNA sequence of 11.0-kb dsRNA (ThHV1) in mycelia of both T-70 and T-70D was obtained by assembling all RT-PCR amplicons and cDNA clones for 5′- and 3′-termini. The assembled full length cDNA sequence of ThHV1 was 11214 bp in length, excluding the 3′-terminal poly A tail of 37 bp in length (GenBank accession No. MN172262). Two ORFs, i.e., ORF1 and ORF2, were deduced from the genome of ThHV1, and the stop codon of ORF1 overlapped with the start codon of ORF2 in a 43 nt long sequence at positions 2248–2291 (Figure 1B). Two untranslated regions (UTRs) located at the 5′- and 3′-termini were 959 nt and 586 nt in length, respectively (Figure 1B). Moreover, two H-type pseudoknot structures were detected in the overlap region of ORF1 and ORF2 at positions 2244–2275 (ΔG value of −22.3 kcal/mol) and positions 2271–2298 (ΔG value of −12.19 kcal/mol) (Figure S2). ORF1 was predicted to encode a polypeptide of 443 aa with a molecular mass of 50.23 kDa. No significant homologous protein was found in the ORF1-encoded polypeptide based on BLSATp search in NCBI database. ORF2 was deduced to encode a polypeptide of 2793 aa with a molecular mass of 319.67 kDa. A sequence analysis of the ORF2-encoded polypeptide revealed that it contained a UDP-glucose/sterol glucosyltransferase (UGT) domain, a putative domain of permuted papain fold peptidases (PPPDE), an RNA-dependent RNA polymerase (RdRp) domain and an RNA helicase (Hel) domain (Figure 1). BLSATp search on NCBI indicated that the ORF2-encoded polypeptide was similar to the polypeptides encoded by viruses in the family *Hypoviridae*, including Botrytis cinerea hypovirus 1 (BcHV1/HBTom-372) (55.3% aa identity), Valsa ceratosperma hypovirus 1(VcHV1/MVC86) (54.7% aa identity), Phomopsis longicolla hypovirus 1 (PlHV1/ME711) (50.4% aa identity), Cryphonectria hypovirus 3 (CHV3/GH2) (55.7% aa identity), Cryphonectria hypovirus 4 (CHV4/SR2) (47.9% aa identity), and Sclerotinia sclerotiorum hypovirus 1 (SsHV1/SZ150) (46.0% aa identity) (Table S2).

The 9816-bp-long (excluding terminal poly A tail of 52 bp in length, GenBank accession No. MN172263) cDNA sequence of ThHV1-S was obtained by assembling the random-primer amplified cDNA clones (Figure 1, Figure S1), specific primer pairs amplified cDNA clones, and the cDNA clones for 5′- and 3′-terminal sequences (Figure S1). The alignment of the genome sequences of ThHV1 and ThHV1-S showed that two regions, i.e., a 1246-nt region I (1110–2357) and a 155-nt region II (10,631–10,785), were missing in the genome of ThHV1-S (Figure S3A). Except for these two missing regions, the nucleotide sequences of the remaining three regions of ThHV1 were 99.95% identical to the full-length sequence of ThHV1-S. The deleted 1246-nt region I in ThHV1-S included almost the entire ORF1 and a tiny proportion of ORF2 of ThHV1, and the putative ORF S in ThHV1-S included the remaining sequences of both ORF1 and ORF2 of ThHV1, whereas the deletion of 155-nt region II in ThHV1-S was located at the 3′-UTR region of ThHV1. The ORF S encoded polypeptide was 2807 aa in length with a molecular mass of 321.41 kDa, containing all four conserved domains in the ORF 2-encoded polypeptide. Except for the deleted region, the amino acid sequence of the ORF S-encoded polypeptide was 100% identical to the ORF2-encoded polypeptide, although a few nucleotides between ThHV1 and ThHV1-S were different. The deletion of region I in ThHV1-S was confirmed using RT-PCR with primer pairs C1F/C1R and C2F/C2R. By using primer pair C1F/C1R, only a 1.6-kb-long DNA band was amplified from strain T-70. However, besides the 1.6-kb-long DNA band, a 400-bp-long DNA band was also amplified from strain T-70D (Figure S3B). For primer pair C2F/C2R, a 500-bp-long DNA band, indicating the presence of ThHV1, was amplified from both strain T-70 and T-70D, as the primer pair C2F/C2R was located in the region absent in ThHV1-S. The deletion of region II in ThHV1-S was also confirmed using RT-PCR with primer pair C3F/C3R, and only one 700-bp-long DNA band was amplified from strain T-70, whereas two DNA bands, of 700 bp and 500 bp in length corresponding to ThHV1 and ThHV1-S respectively, were amplified from strain T-70D (Figure S3C).

In addition, the northern blot analysis was used to confirm the authenticity of the cDNA sequence of ThHV1 and ThHV1-S, and the deleted region I in ThHV1-S. Probe 1 corresponded to the region absent in the ThHV1-S sequence hybridized only with dsRNA of ThHV1, but not with the dsRNA of ThHV1-S (Figure 1C), whereas probe 2 corresponded to the non-deleted region in ThHV1 and ThHV1-S hybridized with both dsRNAs of ThHV1 and ThHV1-S (Figure 1C).

### 3.2. Phylogenetic Analysis

**Figure 2:**
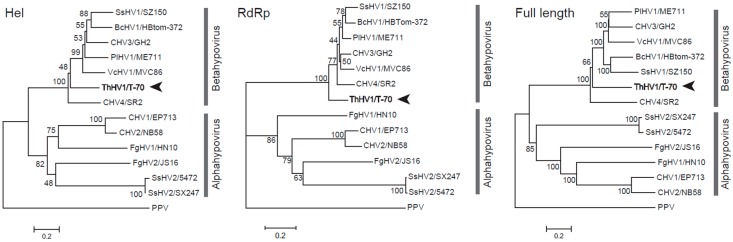
Neighbor-joining trees based on the helicase domain, the RdRp, and the full-length amino acid sequence of ORF2 of Trichoderma harzianum hypovirus 1 (ThHV1) and other hypoviruses. Plum pox virus (PPV) was used as an outgroup in the analyses. The name “ThHV1” was in blod and indicated by arrowheads.

In order to investigate the phylogenetic relationship of ThHV1 with other hypoviruses, three phylogenetic trees based on amino acid sequences of the RdRp domain, Hel domain, and the ORF2-encoded peptide of ThHV1 and other 13 selected RNA viruses were established. Two subclades, i.e., subclade Alphahypovirus and subclade Betahypovirus, with similar topologies were found in all three phylogenetic trees. ThHV1 was clustered with other betahypoviruses in subclade Betahypovirus in all three established trees. In the subclade Betahypovirus of the Hel domain constructed phylogenetic tree, ThHV1 formed an independent branch, whereas virus members CHV3/GH2, CHV4/SR2, SsHV1/SZ150, BcHV1/HBtom-372, VcHV1/MVC86, and PlHV1/ME711 clustered together forming an independent subclade with bootstrap support of 100% (Figure 2). Similarly, the phylogenetic tree constructed using the viral RdRp domain also showed that CHV3/GH2, CHV4/SR2, SsHV1/SZ150, BcHV1/HBtom-372, VcHV1/MVC86, and PlHV1/ME711 firstly formed an independent branch and then clustered with ThHV1, forming the subclade Betahypovirus with a bootstrap of 100% (Figure 2). In the phylogenetic tree constructed by the full length ORF2 encoded polypeptide, ThHV1 also clustered with CHV3/GH2, CHV4/SR2, SsHV1/SZ150, BcHV1/HBtom-372, VcHV1/MVC86, and PlHV1/ME711 forming the subclade Betahypovirus with a bootstrap of 100% (Figure 2).

### 3.3. Debilitated Biological Properties of T. harzianum Isolate T-70D

**Figure 3:**
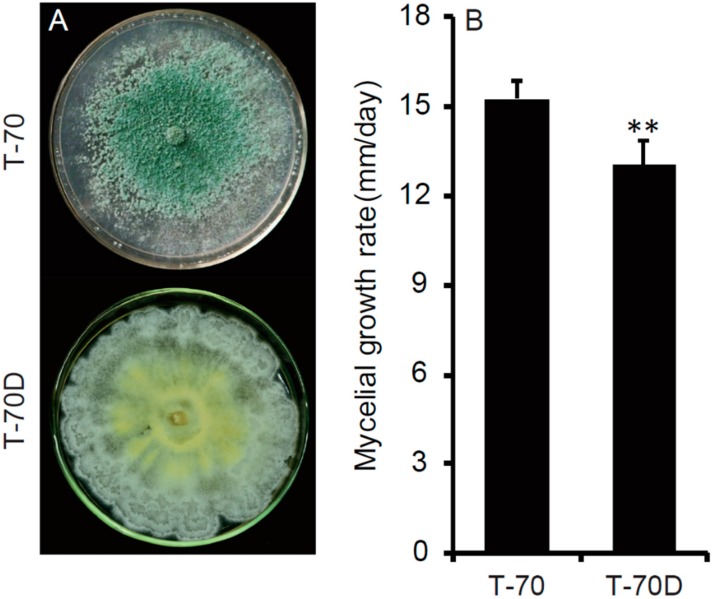
Colony morphology and growth rate of *Trichoderma harzianum* isolates T-70 carrying ThHV1 and T-70D carrying both ThHV1 and ThHV1. (**A**) Colony morphology (20 °C, 10 days). (**B**) Radial mycelial growth rate on potato dextrose agar (*n* = 5); “**” indicates a significant difference between the two isolates according to the Student t test (*p* < 0.01).

After cultivation on PDA plates for 10 days, the colony of isolate T-70D was distinctive in comparison with that of isolate T-70. Isolate T-70D formed abundant mycelial sectors on the colony margin, lacking aerial hyphae and sporulation, and produced orange pigment on PDA plates (Figure 3A). In contrast, isolate T-70 covered the whole petri dish, forming abundant aerial hyphae and green conidia (3.14 × 109 conidia/plate) with no or little pigment formation on the PDA plate (Figure 3A). *T. harzianum* isolate T-70D grew relatively slower on PDA, with an average RGR of 13.1 mm/day, compared to isolate T-70 with an average RGR of 15.2 mm/day (Figure 3B).

**Figure 4:**
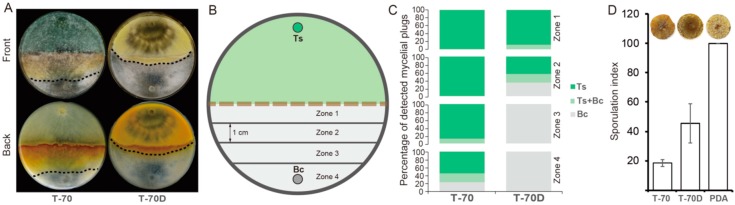
Mycoparasitism ability of *Trichoderma harzianum* isolates T-70 and T-70D against *Botrytis cinerea* in dual cultural assay. (**A**) Colony morphology of dual culture between *T. harzianum* and *B. cinerea* on PDA (20 °C, 7 days). Dashed lines indicate the mycelial interface between *T. harzianum* and *B. cinerea* colonies. (**B**) A schematic diagram showing the four zones (1–4) used for mycelia plugs sampling in a dual culture. The mycelial agar plugs were individually transferred to PDA, and subsequently identified to be colonized either by *T. harzianum* or *B. cinerea*, or by both, based on the distinct colony morphology of the two fungi after incubation at 20 °C for 10 days. (**C**) The frequency of the agar plugs from the four zones of dual culture colonized by *T. harzianum* (dark green) or *B. cinerea* (gray), or both (light green). “Ts” and “Bc” indicate *Trichoderma* spp. and *B. cinerea*, respectively. (**D**) Efficacy of isolates T-70 and T-70D in suppression of sporulation by *B. cinerea* on oilseed rape leaf discs (*n* = 9). A photograph of single leaf disc with sporulation of *B. cinerea* for each isolate was placed on the top of each bar.

The dual-cultural results showed that the mycoparasitic ability of T-70D was significantly decreased in comparison with that of isolate T-70 (Figure 4A). After 10 days, the frequency of *Trichoderma* colonies (both Ts or Ts + Bc) colonized mycelial agar plugs (MAPs) in the four zones (from zone 1 to zone 4) were 100, 100, 100, and 77.8%, respectively, in the dual cultures of isolate T-70 + Bc, but only 100, 66.7, 0 and 0%, respectively, in the dual cultures of T-70D + Bc (Figure 4C).

Strain T-70D grew more slowly than isolate T-70 in PDB. The mycelial dry weight of T-70D was 0.34 g, i.e., significantly lower than 0.57 g of T-70 (Figure S4). Although the pH values of strains T-70 and T-70D were indistinguishable, the PDB cultural filtrate of T-70D showed higher levels of antifungal activity than that of isolate T-70 (Figure S4). The cultural filtrate of T-70 inhibited mycelial growth of *B. cinerea* by only 13.4%, compared to 50.2% inhibition by cultural filtrate of T-70D (Figure S4). However, inconsistent with the growth inhibition by cultural filtrates, isolate T-70 inhibited sporulation of *B. cinerea* on rapeseed leaf disc with a sporulation index of 18, i.e., significantly lower than those of isolate T-70D (46) and the water control (100) (Figure 4D), indicating the stronger ability of isolate T-70 to suppress the sporulation of *B. cinerea* than that of isolate T-70D.

### 3.4. Accumulation of ThHV1 and ThHV1-S

**Figure 5:**
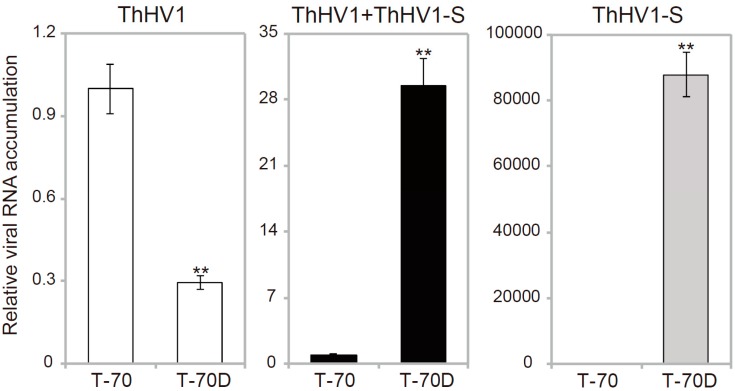
Relative viral RNA accumulation of Trichoderma harzianum hypovirus 1 (ThHV1) and ThHV1-S in *T. harzianum* isolates T-70 and T-70D. “**” indicates a significant difference between the two isolates according to the Student t test (*p* < 0.01).

Accumulation of ThHV1 and ThHV1-S in the *T. harzianum* isolates T-70 and T-70D was determined by semi-quantitative RT-PCR. The average relative accumulation (RA) values of ThHV1 in isolate T-70 was assigned as 1.0, and the RA of ThHV1 in isolate T-70D was slightly decreased compared with that of isolate T-70, with a value of 0.29 (see Figure 5). However, the average RA total value of both ThHV1 and ThHV1-S was dramatically increased to approximately 29.4 times that of ThHV1 in isolate T-70. No accumulation of ThHV1-S in isolate T-70 was detected (see Figure 5).

### 3.5. Vertical Transmission of ThHV1 and ThHV1-S

More than 100 single-conidium progenies were obtained from isolate T-70D; they all showed similar cultural morphology to their parental isolate T-70D, including forming colony margin sectors, lacking aerial hyphae, slow radial mycelial growth, and rare sporulation. The RT-PCR detection showed that ThHV1 and ThHV1-S are present in all 10 random selected single-conidium progenies of isolate T-70D (Figure S5). These results suggest that ThHV1 and ThHV1-S in strain T-70D could be vertically transmitted through conidia.

### 3.6. Effects of ThHV1 and ThHV1-S on Bioligical Properties of T. harzianum and T. koningiopsis

**Figure 6:**
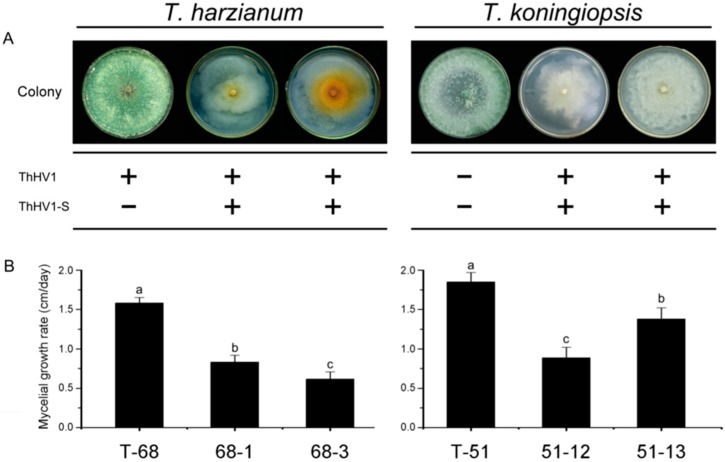
Impact of Trichoderma harzianum hypovirus 1 (ThHV1) and ThHV1-S infection on T*. harzianum* isolate T-68 and *T. koningiopsis* isolate T-51. (**A**) Colony morphology on PDA and the presence of ThHV1 and ThHV1-S in different *Trichoderma* isolates/strains. The symbols “+” and “−” indicate the presence and the absence of the indicated viruses, respectively, according to the detection by RT-PCR. (**B**) Radial mycelial growth rate on PDA of different *Trichoderma* isolates/strians (*n* = 9). Bars labeled with the same letters are not significantly different (*p* < 0.05) according to Least Significant Difference Test.

**Figure 7:**
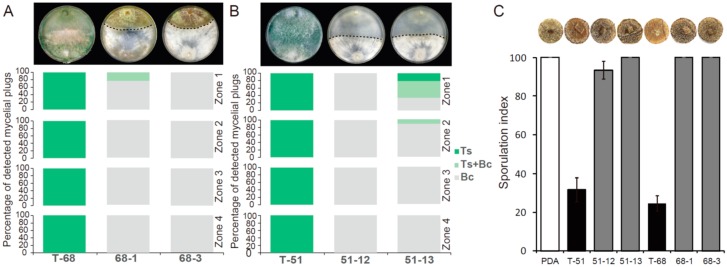
Mycoparasitism ability against *Botrytis cinerea* and suppression of *B. cinerea* sporulation for *Trichoderma harzianum* isolate T-68, *T. koningiopsis* isolate T-51 and their derivative strains. The duel culture morphology and the photograph of single leaf disc with *B. cinerea* sporulation for each isolate/strain of *Trichoderma* spp. was placed on the top of the corresponding bar of each isolate/strain. (**A**) The frequency of agar plugs from the four zones of dual culture against *B. cinerea* for isolate T-68 and its derivative strains colonized by *Trichoderma* spp. (dark green) or *B. cinerea* (gray), or both (light green). (**B**) The frequency of agar plugs from the four zones of dual culture against *B. cinerea* for isolate T-51 and its derivative strains colonized by *Trichoderma* spp. or *B. cinerea*, or both. (**C**) Suppression of *B. cinerea* sporulation on oilseed rape leaf discs by different *Trichoderma* spp. isolate/strain.

In the horizontal transmission of both ThHV1 and ThHV1-S from T-70D, four mycelial derivative strains were established after 10 days of dual culture by picking up the mycelial plugs from the colonies of *T. harzianum* isolate T-68 and *T. koningiopsis* isolate T-51. Two derivative strains from isolate T-68, namely 68-1 and 68-3, as well as two derivative strains from isolate T-51, namely 51-12 and 51-13, were used for further characterization (Figure 6). ThHV1 and ThHV1-S were detected in all four derivative strains through RT-PCR (Figure S6). These derivative isolates showed apparently different colony morphology by comparison with their parental isolates. After cultivation on PDA for 10 days, the parental isolate T-68 produced mass conidial clusters and aerial hyphae, whereas its derivative strains 68-1and 68-3 rarely produced conidial cluster and aerial hyphae (Figure 6). Isolate T-68 grew rapidly on the PDA plate with an average RGR of 15.8 mm/day, while the derivative strains 68-1 and 68-3 grew slowly, with an average RGR of 8.3 and 6.1 mm/day, respectively (Figure 6). Similar phenotypic changes were also observed on derivative strains 51-12 and 51-13 carrying both ThHV1 and ThHV1-S from *T. koningiopsis* isolate T-51. The colony morphologies of 51-12 and 51-13 were abnormal, with the rare formation of conidial clusters (Figure 6). The RGR of 51-12 and 51-13 were 8.9 and 13.7 mm/day, respectively, which were significantly decreased in comparison with their parental isolate T-51 (18.5 mm/day) (Figure 6). Moreover, the mycoparasitism assay showed that the mycoparasitic abilities of the four derivative strains dramatically decreased by comparing with their respective parental isolates T-51 and T-68. The average frequency of *T. harzianum* isolate T-68 colonized MAPs (Ts alone and Ts + Bc) in all four zones was 97.2%, whereas the frequency of its derivative strains 68-1 and 68-3 colonized MAPs were only 5.6 and 0%, respectively, in the dual cultures of *T. harzianum* + Bc (Figure 7A). Similar results were also observed in the derivative strains from isolate T-51, only 0 and 19.4% MAPs for the derivative strains 51-12 and 51-13, respectively, were colonized by *T. koningiopsis*, whereas the frequency of the parental isolate T-51 colonized MAPs was 100%, in dual cultures of *T. koningiopsis* + Bc (Figure 7B). These results indicated a devastating decline of mycoparasitic ability of *T. harzianum* isolate T-68 and *T. koningiopsis* isolate T-51 accompanied with the infection of both ThHV1 and ThHV1-S. However, the two strains 51-70-2 and 51-70-4 derived from isolate T-51 after dual culture with isolate T-70 carrying ThHV1 alone showed no significant changes in biological properties, including colony morphology and mycoparasitic ability, from those of their parental isolate, i.e., T-51 (Figure S9). These results indicate that infection by ThHV1 alone had no observable effect on *T. koningiopsis*.

The inhibitions of *B. cinerea* sporulation by different *Trichoderma* isolates/strains were determined on leaf discs. *B. cinerea* sporulated profusely on control leaf discs with an average sporulation index of 100. In contrast, *Trichoderma* isolates T-68 and T-51 significantly reduced the sporulation, with average indices of 24 and 32, respectively. However, all four derivative strains, i.e., 68-1, 68-3, 51-12, and 51-13, were less effective than their respective parental strains in inhibiting sporulation of *B. cinerea*, with sporulation indicies of 100, 100, 93, and 100, respectively. Thus, compared with control treatment, isolates T-68 and T-51 suppressed *B. cinerea* sporulation by 76% and 68%, respectively, whereas strains 68-1, 68-3, 51-12, and 51-13 suppressed *B. cinerea* sporulation by only 0%, 0%, 7%, and 0%, respectively (Figure 7C).

### 3.7. Effects of ThHV1 and ThHV1-S on Antifungal Activities of T. harzianum and T. koningiopsis

Similar to the donor strain T-70D, the cultural filtrates of the four derivative isolates showed significantly higher levels of antifungal activities than their respective parental isolates (Figure S7). The cultural filtrate of isolate T-68 inhibited *B. cinerea* growth by was 44.3%, while the cultural filtrates of two derivative strains 68-1 and 68-3 inhibited *B. cinerea* growth by 63.8 and 63.3%, respectively (Figure S7). No significant difference was observed in the cultural pH value and mycelial dry weight among T-68, 68-1, and 68-3 (Figure S7). For the isolate T-51 of *T. koningiopsis*, the cultural filtrate of isolate T-51 inhibited *B. cinerea* growth by 64.9%, whereas the cultural filtrates of the two derivative strains, i.e., 51-12 and 51-13, inhibited the *B. cinerea* growth rate by 75.8% and 98.1%, respectively (Figure S7). No significant difference in mycelial dry weight was observed among T-51, 51-12, and 51-13, but the cultural pH values of strains 51-12 and 51-13 were significantly increased in comparison with that of isolate T-51 (Figure S7). In *in vivo* pathogenicity trials, *B. cinerea* infected tomato leaf, causing lesions of 1.98 cm in diameter. The lesion diameter on tomato leaflets with the *T. harzianum* isolates T-68 and the two derivative strains 68-1 and 68-3 cultural filtrate were 1.66 cm, 1.63 cm, and 1.49 cm, respectively, which were not significantly different (Figure S8). For *T. koningiopsis* isolate T-51 and the two derivative strains, i.e., 51-12 and 51-13, the lesion diameter on tomato leaf were 1.34 cm, 1.12 cm, and 0.62 cm, respectively, all of which showed significant differences compared with the control leaf; moreover, inhibition by strain 51-13 showed a significant increase compared with the original isolate T-51(Figure S8).

### 3.8. Incidence and Distribution of ThHV1

In order to investigate the incidence and distribution of ThHV1 in China, 36 *Trichoderma* isolates obtained from soil in China were tested for the presence of both ThHV1 and ThHV1-S. ThHV1 infection was detected in 6 out of the 36 (16.7%) tested *Trichoderma* isolates (Table S3), while ThHV1-S was not detected in all isolates. Besides *T. harzianum*, ThHV1 was also detected in *T. koningiopsis* and *T. atroviride* isolates obtained from field soil. In these ThHV1-infected strains, *T. koningiopsis* T-35, *T. koningiopsis* T-37 and *T. atroviride* T-38 were collected from the same location (Wuhan, China), whereas *Trichoderma* sp. JST-12 and *Trichoderma* sp. YN8-2 were collected from Hangzhou in Zhejiang Province and Xinyang in Henan Province, respectively.

## 4. Discussion

This study molecularly characterized a novel hypovirus and its D-RNA, namely ThHV1 and ThHV1-S, from the *T. harzianum* isolate T-70D, and established the association of ThHV1-S with the debilitation of isolate T-70D, including impaired mycelial growth and a significant reduction of mycoparasitic ability against *B. cinerea*. The genome of ThHV1 is 11214 nts long, excluding the poly (A) tail, containing two overlapping ORFs (ORF1 and ORF2), and the genome of ThHV1-S is 9816 nts in length without a poly (A) tail, containing only one large ORF (ORF S). A BLAST search and sequence alignment of the ORF2-encoded polypeptide indicated that ThHV1 might be a member of the *Hypoviridae* viral family (Figure 2).

So far, two (Alphahypovirus and Betahypovirus) or three (Alphahypovirus, Betahypovirus and Gammahypovirus) genera have been suggested in the family *Hypoviridae* [19,36,37,38]. Overall, alphahypoviruses, including CHV1/EP713, CHV2/NB58, and FgHV1/HN10, possess two ORFs, of which the larger ORF contains three conserved domains (Prot, RdRp and Hel). Betahypoviruses, including CHV3/GH2, CHV4/SR2, PIHV1, SsHV1/SZ150, and VcHV1/MVC86, contain only one ORF encoding a single polyprotein with four conserved domains (Prot, RdRp, Hel, and UGT) [37]. The gammahypovirus accommodates only one viral member, i.e., SsHV2, and is phylogenetically related to alphahypoviruses, but it possesses only one ORF that encodes a single polyprotein with the same conserved domains found in alphahypoviruses [36]. Our results showed that the genome size of ThHV1 was 11.2 kb, which is in the range of genome size (9 to 13 kb) for vast majority of hypoviruses. Moreover, the ThHV1 ORF2-encoded polypeptide was more similar to those of betahypoviruses, and contained the conserved domain UGT that is not present in genomes of alphahypoviruses. In addition, phylogenetic analyses based on the polyprotein and conserved domains (RdRp and Hel) clearly showed that ThHV1 was clustered with members of betahypovirus. However, the genomic structure of ThHV1 was apparently different from those of the betahypoviruses, as ThHV1 possesses two overlapping ORFs, whereas betahypoviruses possess only one. It is interesting that the expression strategy of the two ORFs in ThHV1 was also different from alphahypoviruses. The two ORFs in alphahypoviruses overlap in the pentanucleotide UAAUG, whereas the two ORFs in ThHV1 overlapped in the 44 nt sequence. A potential pseudoknot structure was detected in the 44 nt overlapping sequence, and a similar structure was also detected in rat ornithine decarboxylase antizyme involving in an autoregulatory +1 frameshifting [39]. Therefore, the pseudoknot structure detected in the overlap region between ORF1 and ORF2 may be responsible for a +1 frameshifting. In this case, instead of forming two polypeptides encoded by ORF1 and ORF 2 respectively, ORF2 is more likely expressed as a fusion protein with ORF1 formed by a +1 frameshifting in the 44 nt overlap region between the two ORFs. Consequently, ThHV1 may be a novel member in the family of *Hypoviridae*, and may belong to a new viral genus.

D/DI-RNAs are a type of subviral RNA found in many RNA viruses, and their replication is completely dependent on enzymes encoded by their helper viruses. D/DI-RNAs are also recorded in hypoviruses including CHV1/EP713, CHV3/GH2, and FgHV2/JS16, and some of them encode truncated proteins [40,41,42]. Similar to D-RNAs found in CHV3/GH2 and FgHV2/JS16, ThHV1-S, the D-RNA of ThHV1 also possesses a single large ORF encoding a polypeptide containing a major portion of ORF2-encoded polypeptide fused with a tiny portion of ORF1-encoded polypeptide. As the genome of ThHV1-S possesses all four conserved domains, especially the RdRp domain, it is noteworthy that ThHV1-S could replicate using proteins encoded by itself, without the help of the parental virus (ThHV1). More interestingly, the genomic structure of ThHV1-S is more similar to those of betahypoviruses, as all of their genomes possess only one large ORF. To test whether ThHV1-S could replicate independently, single-conidium strains of isolate T-70D were established to search the strain carrying ThHV1-S alone, but all of these strains were determined to be infected by both ThHV1 and ThHV1-S. Therefore, further studies to elucidate the replication strategy of ThHV1-S are warranted.

Although most mycoviruses do not cause any visible symptoms to their host fungi, some mycoviruses do affect the biological properties of the host, especially for the hypovirulence-associated mycoviruses infecting plant pathogenic fungi [13]. In this study, significant differences on biological properties were observed between strains T-70 and T-70D, including mycelial growth, conidial production, mycoparasitic ability, and antifungal ability. To elucidate the effect of ThHV1 and ThHV1-S on the phenotypic changes of strain T-70D, many methods were exploited to cure the virus in strains T-70 or T-70D, including picking hyphal tip, anti-viral chemical treatment, and protoplast regeneration. However, no ThHV1-free strain was obtained. On the other hand, ThHV1 and ThHV1-S were transmitted to isolates T-68 of *T. harzianum* and T-51 of *T. koningiopsis*. The derivative strains 68-1 and 68-3 of *T. harzianum*, and strains 51-12 and 51-13 of *T. koningiopsis*, carrying both ThHV1 and ThHV1-S, also showed significant decreases in mycelial growth, conidial production, and mycoparasitic ability compared with their corresponding parental strains T-68 and T-51, respectively (Figure 7). Although higher antifungal activities were observed in the derivative strains carrying both ThHV1 and ThHV1-S (Figure S7), these strains showed less inhibition of *B*. *cinerea* sporulation on leaf discs, indicating that these strains may still display less biocontrol efficiency. In contrast, derivative strains 51-70-2 and 51-70-4, carrying ThHV1 alone, showed no observable changes in colony morphology and mycoparasitic ability in comparison with their parental isolate T-51. These findings indicate close associations of ThHV1-S with the decline of strain T-70D and the derivative isolates 68-1, 68-3, 51-12, and 51-13. The reduction of conidial production in ThHV1-S-infected *Trichoderma* spp. may present a problem during the commercial production of *Trichoderma*. A previous study showed the presence of mycovirus in some mycoparasites, for instance the victorivirus, namely Coniothyrium minitans RNA virus (CmRV), was detected in *C. minitans*, a mycoparasite of *S. sclerotiorum* [43]. However, CmRV seems to have no apparent effect on its host. In addition, dsRNAs have also been detected in some populations of *Trichoderma* spp., as a causing agent of green mold disease in mushrooms in Korea [44]. Recently, more mycoviruses have been detected in *T. atroviride* [45,46], *T. harzianum* [46], and *T. asperellum* [47]. Within these reports, one research showed that infection by Trichoderma harzianum partitivirus 1 (ThPV1) may increase the antifungal activity of *T. harzianum* [46]. However, the effects of ThPV1 infection on the biocontrol efficacy of *T. harzianum* remains unknown. To our knowledge, this study is the first to show that the coinfection of a mycovirus (ThHV1) and its D-RNA (ThHV1-S) in a mycoparasitic fungus results in a significant decrease of mycoparasitic ability.

In some cases, D-RNAs are termed DI-RNAs, i.e., if they are able to suppress helper virus accumulation and attenuate disease symptoms induced by their helper viruses [20]. For instance, DI-RNAs of Rosellinia necatrix partitivirus 2 (RnPV2) have been shown to be able to reduce the RnPV2 accumulation and the symptoms caused by RnPV2 in a Dicer-like 2 knockout mutant of *C. parasitica* [21]. Similarly, the accumulation of helper virus ThHV1 in isolate T-70D was decreased by one-third of the expression level of ThHV1 in isolate T-70; this may be due to the interference of ThHV1-S. However, compared with isolate T-70, the total accumulation of both ThHV1 and ThHV1-S was increased by almost 30 times in T-70D. Therefore, this may be responsible for the decline of mycelial growth and mycoparasitic ability against *B. cinerea* in isolate T-70D.

Compared with chemical control, the efficacy and consistency of biological control was commonly affected by many factors, mainly due to its dependence on living organisms. The effects of environmental parameters, such as temperature, moisture, soil type, host cultivar, and others could affect biocontrol performance [48]. Although the antifungal activity of *Trichoderma* isolates carrying both ThHV1 and ThHV1-S increased, some other properties associated with the biocontrol efficacy of *Trichoderma* spp. were severely impaired. Therefore, the present study demonstrated that viruses in fungal BCA may also be a factor associated with the stability of biocontrol in fields. Firstly, *Trichoderma* isolates/strains showed a decrease of mycoparasitic ability when infected with ThHV1-S, indicating that their mycoparasitism on fungal pathogens may decrease in the field as well. Secondly, the suppression of *B. cinerea* sporulation was severely decreased in ThHV1-S-infected *Trichoderma* strains/isolates, indicating that ThHV1-S infection may decrease the competition of *Trichoderma* with other fungal pathogens in the field. Sporulation is essential for the epidemic of *B. cinerea*, and the unsuccessful suppression of *B. cinerea* sporulation can lead to failed control of *B. cinerea* in the field. Finally, it is noteworthy that ThHV1 and ThHV1-S can be transmitted from isolate T-70D, not only to another isolate of the same species, but also to an isolate of a different species (*T. koningiopsis* isolate T-51). Moreover, RT-PCR detection indicated that ThHV1 might be present in three species of *Trichoderma* (*T. harzianum*, *T. koningiopsis* and *T. atroviride*) with an incidence of approximately 16.7%. Therefore, ThHV1 may have a wide geographic distribution in China (Table S3). However, there is one weak point that the amplicons should be sequenced in order to confirm the presence of ThHV1. These observations suggest that ThHV1 and ThHV1-S have a strong potential to spread among populations of different *Trichoderma* spp. in soils, as some previous research has suggested that viruses can be acquired during fungal vegetative growth under natural conditions without the limitation of vegetative incompatibility [49,50]. This may have a great impact on using *Trichoderma* spp. as a biocontrol agent if ThHV1 or ThHV1-S persists. As ThHV1 infection alone seems to have no apparent effect on its host, the decline of certain *Trichoderma* spp. may occur once the ThHV1-S was generated. Nonetheless, *Trichoderma* spp. were also well known for their ability to produce a wide range of antibiotic substances [51], and the antifungal activity of the derivative *Trichoderma* strains was increased, especially in strain 51-13. The higher antifungal activities of the derivative strains carrying both ThHV1 and ThHV1-S (Figure S7) may be due to the effects of viral infection on the production of second metabolites, as some viral infections were able to increase [16] or decrease [52] the production of secondary metabolites in fungi. Although we could not rule out the possibility that the ThHV1- or/and ThHV1-S-encoded proteins may show antifungal activity, considering that non-viral protein secretion was predicted in the fungal hosts, we prefer the former explanation. This may also indicate that mycovirus have the potential to improve the production of certain secondary metabolites, and be further used to engineer *Trichoderma* strains for antifungal compound production.

## 5. Conclusions

The results of present study showed that ThHV1 is a novel hypovirus, probably belonging to a novel viral genus. Infection of both ThHV1 and ThHV1-S, not ThHV1 alone, resulted in the abnormal biological properties of *Trichoderma* spp., especially decreased mycoparasitism ability and inhibition of *B*. *cinerea* sporulation. In addition, ThHV1 and ThHV1-S could be horizontally transmitted from *T*. *harzianum* isolate T-70D to both *T*. *harzianum* isolate T-68 and *T*. *koningiopsis* isolate T-51. ThHV1 had a relatively wide geographic distribution in China, naturally infecting the population of *T*. *harzianum*, *T*. *koningiopsis* and *T*. *atroviride*. Therefore, the present study demonstrated that mycoviruses could shape the interactions between mycoparasites and their host fungi, and may be a factor responsible for the unstable field performance for many fungal biocontrol agents.

## Figures and Tables

**Table 1:** Origin of *Trichoderma* strains/isolates used in this study.

Strains	Origin (Place and Collected Time)	Presence of Virus	
		ThHV1	ThHV1-S
**Field strains**			
T-70	Ezhou, China, 2012	+ ^1^	–
T-68	Ezhou, China, 2012	+	–
T-51	Wuhan, China, 2012	–	–
**Laboratory-Derived Strains**			
T-70D	colony edge progeny of T-70, 2013	+	+
68-1	T-68 in a pairing-culture of T-68 and T-70D, 2016	+	+
68-3	T-68 in a pairing-culture of T-68 and T-70D, 2016	+	+
51-12	T-51 in a pairing-culture of T-51 and T-70D, 2016	+	+
51-13	T-51 in a pairing-culture of T-51 and T-70D, 2016	+	+
51-70-2	T-51 in a pairing-culture of T-51 and T-70, 2016	+	–
51-70-4	T-51 in a pairing-culture of T-51 and T-70, 2016	+	–

^1^ The symbols “+” and “–” indicate the presence and absence of ThHV1 or ThHV1-S, respectively.

## Supplementary Materials

The following are available online at https://www.mdpi.com/2076-2607/7/11/507/s1.
