# Supplementary material for: Defective RNA of a Novel Mycovirus with High Transmissibility Detrimental to Biocontrol Properties of Trichoderma spp
Source: Microorganisms. 2019 Oct 29;7(11):507. doi: 10.3390/microorganisms7110507 (PMC6920978; doi:10.3390/microorganisms7110507)
Supplement: Supplementary file 1 [file microorganisms-07-00507-s001.pdf]

microorganisms

MDPI

Supplementary Materials

Table S1. Primers used in this study.

Primer name	Sequence (5′→3′)	Position [1]		Polarity [2]
cDNA cloning of ThHV1 and ThHV1-S		ThHV1	ThHV1-S	
G1F	TTAGCAAGAGCACCAAAT	5389–5406	4143–4160	+
G1R	CAATGAGCCAATAACAGG	6130–6147	4884–4901	–
G2F	AGCATTTAGCTTATCGGCATCA	7667–7688	6421–6442	+
G2R	TGGGCACAGTTCATTTGTAGAG	8324–8345	7078–7099	–
G3F	CGGTTTGCTGGAGATAG	8637–8653	7391–7407	+
G3R	ATACGAACACTGGGTAGAA	9875–9893	8629–8647	–
P1F	AGTCGTGCTGGTTCTAAAAT	5878–5897	4632–4651	+
P1R	GTCACTGGGCTTTTCTCTAA	6374–6393	5218–5147	–
P5F	AAATGATTGTGAGGTCGTA	3633–3651	2387–2405	+
P5R	ATTCGGTCGGTTGTGA	5576–5591	4330–4345	–
P6F	AATTACCTGCGCTTGGGACA	5957–5976	4711–4730	+
P6R	GGAAGCATGGCCTGTTGTTG	7892–7911	6646–6665	–
P7F	CTGGAATCGCTAGCCGAGAA	707–726	707–726	+
P7R	ACTGCCATCAGACCACCTTG	3820–3829	2574–2593	–
70-5SP	CCAACAGTCTGCCATTCCACC	2614–2634	1368–1388	–
70-3SP	GGGGCTGTGCCACGAAA	10269–10285	9023–9039	+
2012-5SP	GGGGGTTGCCCTAGTAAAGA	860–879	860–879	–
2012-3SP	ATGAACTTTATGAAGACGGACTC	9641–9663	8395–8417	+
110A (adapter)	TATCTTATCGGCGTGTCCCCC	to 5′ and 3′-end of dsRNA		+/–
RC110A (primer)	GGGGGACACGCCGATAAGATA	complementary to the adapter 110A		–/+
To detect the presence of ThHV1 and ThHV1-S		ThHV1	ThHV1-S	
C1F	TTGTTGTTTTACCCGCCCCT	979–998	979–998	+
C1R	GGCCTCATGTTCTGGTCGAA	2585–2604	1339–1358	–
C2F	GAACGAGGGGAAGGTGTCAG	1647–1666	/	+
C2R	GCGCTTGTGACCATTAACCC	2107–2126	/	–
C3F	GCCTCATTATCGAGGTTTAG	10507–10526	9261–9280	+
C3R	CAGTATTCGCAGTGCTGTT	11094–11112	9693–9711	–
For semi-quantitative PCR detection of ThHV1 and ThHV1-S				
TML-F	GGTATGACGGTGAAGTGT	1570–1587	/	+
TML-R	ATCAAGAGGAGACCCAAT	1710–1727	/	–
TMS-F	ACCAAACAGGGAAGGACG	1073–1090	1073–1090	+
TMS-R	CTTGTGGCATAACGAACC	2471–2488	1225–1242	–
TMLS-F	ACCGTAAGTTTATTCAGCA	8423–8441	7174–7192	+
TMLS-R	CTCAAAGTATCCCACCAG	8559–8576	7310–7327	–
Tubulin-F	CCAAGCTCTTGTCCTGCCA	/	/	+
Tubulin-R	CAATCTCACGCATGATGGCT	/	/	–
To test cDNA inserts in the pMD18-T vector				
M13F-47	CGCCAGGGTTTTCCCAGTCACGAC	pMD18-T vector		
M13R-48	AGCGGATAACAATTTCACACAGGA	pMD18-T vector		

[1] Positions of oligonucleotides for primers or the adapter in the cDNA of ThHV1/T-70 and ThHV1-S/T-70D were labeled in Figure S1 of this paper. [2] Polarity refers to positive strand (+) and negative strand (–) of dsRNA.

Table S2. Sequence identities between ThHV1 and other hypoviruses.

Family	Genus	Virus	Acronym	Amino Acid Identities (%)					Accession NO.
				ORF2 Full	UGT	PPPDE	RdRp	Hel	
Hypoviridae	**Betahypovirus**	**Sclerotinia sclerotiorum hypovirus 1**	**SsHV1/SZ150**	45.98	53.10	68.18	76.17	58.40	JF781304
	Alphahypovirus	Phomopsis Longicolla hypovirus 1	PlHV1/ME711	50.43	58.69	67.29	75.00	59.22	KF537784
		Cryphonectria hypovirus 3	CHV3/GH2	55.74	57.55	66.97	73.83	60.39	NP-051710.1
		Botrytis cinerea hypovirus 1	BcHV1/HBTom-372	55.31	54.90	64.42	74.61	59.06	MG554632
		Cryphonectria hypovirus 4	CHV4/SR2	47.86	55.88	67.59	71.48	59.51	YP-138519.1
		Valsa ceratosperma hypovirus 1	VcHV1/MVC86	54.71	56.94	67.59	71.76	60.47	KF537784
		Cryphonectria hypovirus 1	CHV1/EP713	8.22	-	-	24.05	14.56	NP041092.1
		Cryphonectria hypovirus 2	CHV2/NB58	9.04	-	-	23.90	18.75	NP613266.1
		Sclerotinia sclerotiorum hypovirus 2	SsHV2/SX247	8.00	-	-	20.21	18.40	AIA61616.1
		Sclerotinia sclerotiorum hypovirus 2	SsHV2/5472	7.54	-	-	20.21	13.43	AHA56680.1
		Fusarium graminearum hypovirus 1	FgHV1/HN10	7.91	-	-	27.06	23.16	AGC75065.1
		Fusarium graminearum hypovirus 2	FgHV2/JS16	7.27	-	-	11.20	19.90	AKB94065.1
Potyviridae	*Potyvirus*	Plum pox virus	PPV	7.88	-	-	13.56	14.05	NP-040807

Table S3. The presence of Trichoderma harzianum hypovirus 1 (ThHV1) and ThHV1-S in the population of *Trichoderma* spp. in China.

Isolate	Source	Location	Species	ThHV1+[1]	ThHV1-S+
T-9	Soil	Enshi, Hubei	*Trichoderma hamatum*	-	-
T-13	Soil	Enshi, Hubei	*Trichoderma atroviride*	-	-
T-18	Soil	Enshi, Hubei	*Trichoderma hamatum*	-	-
T-19	Soil	Enshi, Hubei	*Trichoderma hamatum*	-	-
T-21	Soil	Wuhan, Hubei	*Trichoderma harzianum*	-	-
T-26	Soil	Wuhan, Hubei	*Trichoderma atroviride*	-	-
T-31	Soil	Wuhan, Hubei	*Trichoderma album*	-	-
T-32	Soil	Wuhan, Hubei	*Trichoderma album*	-	-
T-33	Soil	Wuhan, Hubei	*Trichoderma koningiopsis*	-	-
T-34	Soil	Wuhan, Hubei	*Trichoderma koningiopsis*	-	-
T-35	Soil	Wuhan, Hubei	*Trichoderma koningiopsis*	+	-
T-37	Soil	Wuhan, Hubei	*Trichoderma koningiopsis*	+	-
T-38	Soil	Wuhan, Hubei	*Trichoderma atroviride*	+	-
T-41	Soil	Wuhan, Hubei	*Trichoderma harzianum*	+	-
T-49	Soil	Wuhan, Hubei	*Trichoderma koningiopsis*	-	-
T-50	Soil	Wuhan, Hubei	*Trichoderma polysporum*	-	-
T-52	Soil	Wuhan, Hubei	*Trichoderma polysporum*	-	-
T-57	Soil	Hanchuan, Hubei	*Trichoderma koningiopsis*	-	-
T-69	Soil	Ezhou, Hubei	*Trichoderma brevicompactum*	-	-
JST-1	soil	Nanjing, Jiangsu	*Trichoderma* sp.	-	-
JST-2	soil	Nanjing, Jiangsu	*Trichoderma* sp.	-	-
JST-3	soil	Nanjing, Jiangsu	*Trichoderma* sp.	-	-
JST-4	soil	Nanjing, Jiangsu	*Trichoderma* sp.	-	-
JST-10	soil	Hangzhou, Zhejiang	*Trichoderma* sp.	-	-
JST-11	soil	Hangzhou, Zhejiang	*Trichoderma* sp.	-	-
JST-12	soil	Hangzhou, Zhejiang	*Trichoderma* sp.	+	-
JST-14	soil	Hangzhou, Zhejiang	*Trichoderma* sp.	-	-
YN8-2	Straw of oilseed rape	Xinyang, Henan	*Trichoderma* sp.	+	-
YN8-4	Straw of oilseed rape	Xinyang, Henan	*Trichoderma* sp.	-	-
YN8-49	Straw of oilseed rape	Xinyang, Henan	*Trichoderma* sp.	-	-
3-1-221	Straw of oilseed rape	Zhangzhou, Fujian	*Trichoderma* sp.	-	-

JN8M-2	Straw of oilseed rape	Jinxian, Jiangxi	*Trichoderma* sp.	-	-
JN8M-4	Straw of oilseed rape	Jinxian, Jiangxi	*Trichoderma* sp.	-	-
JN8M-8	Straw of oilseed rape	Jinxian, Jiangxi	*Trichoderma* sp.	-	-
JN8M-9	Straw of oilseed rape	Jinxian, Jiangxi	*Trichoderma* sp.	-	-
EN8-72	Straw of oilseed rape	Hubei	*Trichoderma* sp.	-	-

1 "+" represents the presence of ThHV1 or ThHV1-S through the detection of RT-PCR with primer pairs listed in Table S1.

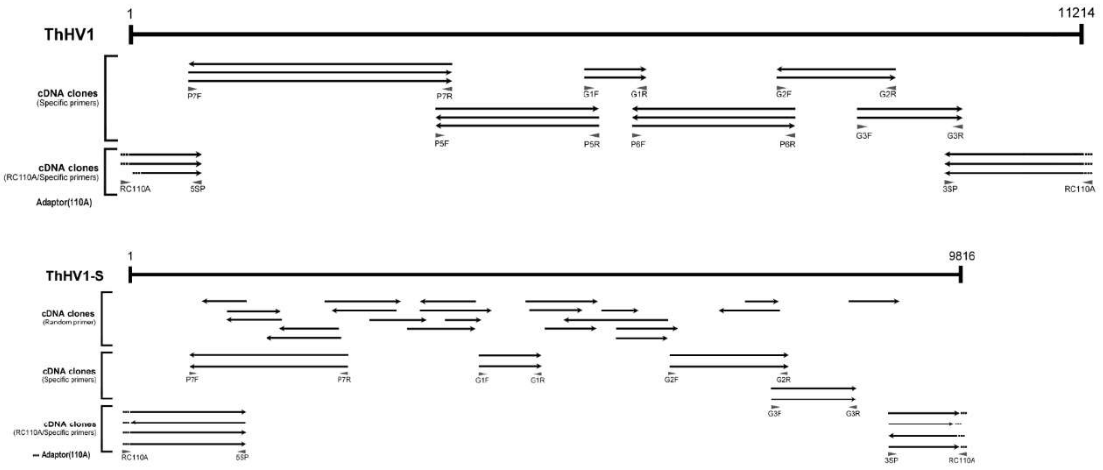

Figure S1. A schematic diagram showing the strategy used for full-length cDNA cloning of Trichoderma harzianum hypovirus 1 (ThHV1) and ThHV1-S. The location of PCR primers and the 5′ and 3′-adaptor used in the cDNA cloning are indicated.

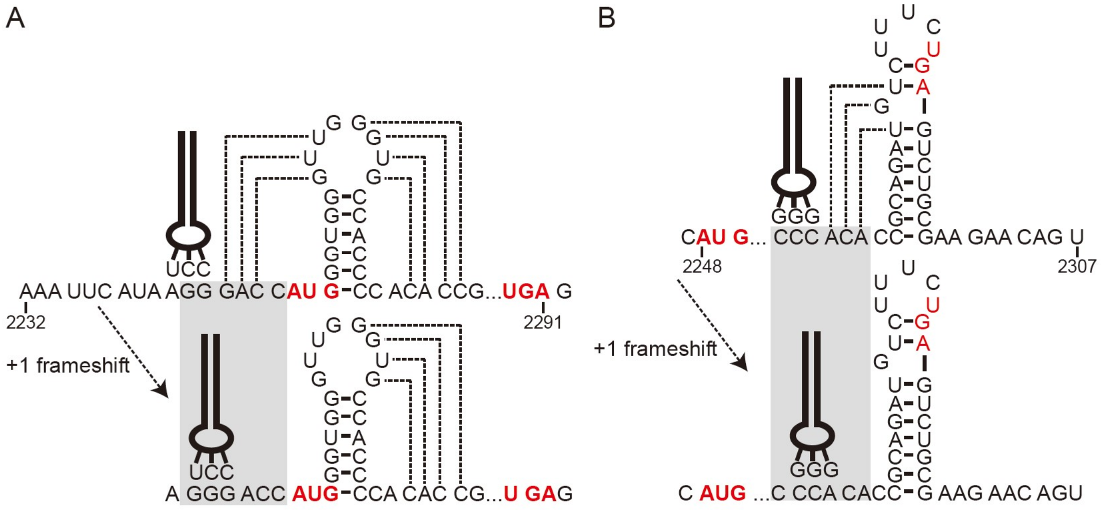

Figure S2. Two possible H-type pseudoknots upstream the overlap region of Trichoderma harzianum hypovirus 1 (ThHV1), and possibly responsible for the +1 frameshift of ThHV1 during the expression of ORF 2 encoded polypeptide. (**A**) A predicted RNA pseudoknot structure is located preceding the start site of ORF2. (**B**) Another predicted RNA pseudoknot structure is located preceding the stop site of ORF1. Dashed lines indicate base pairs predicted to form the stems in the pseudoknots.

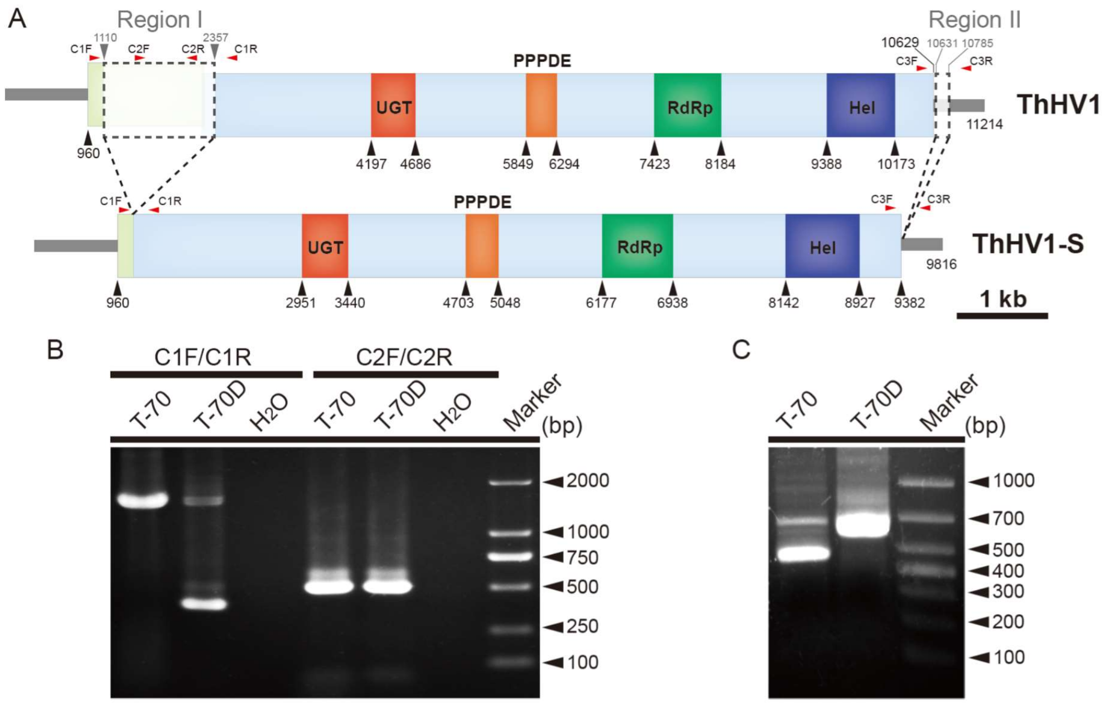

Figure S3. Conformation of the deleted region (I and II) of Trichoderma harzianum hypovirus 1 (ThHV1)-S in comparison with ThHV1 using RT-PCR. (**A**) Schematic diagram showing the deleted regions of ThHV1-S compared with ThHV1. Two dashed line frames indicate the two deleted regions on the genome of ThHV1, and the positions of primers used for RT-PCR detection are indicated as red arrowheads. (**B**) The RT-PCR detection of deleted region I on the genomes of both ThHV1 and ThHV1-S with primer pairs of C1F/C1R and C2F/C2R. C. The RT-PCR detection of deleted region II on the genomes of both ThHV1 and ThHV1-S with primer pair of C3F/C3R.

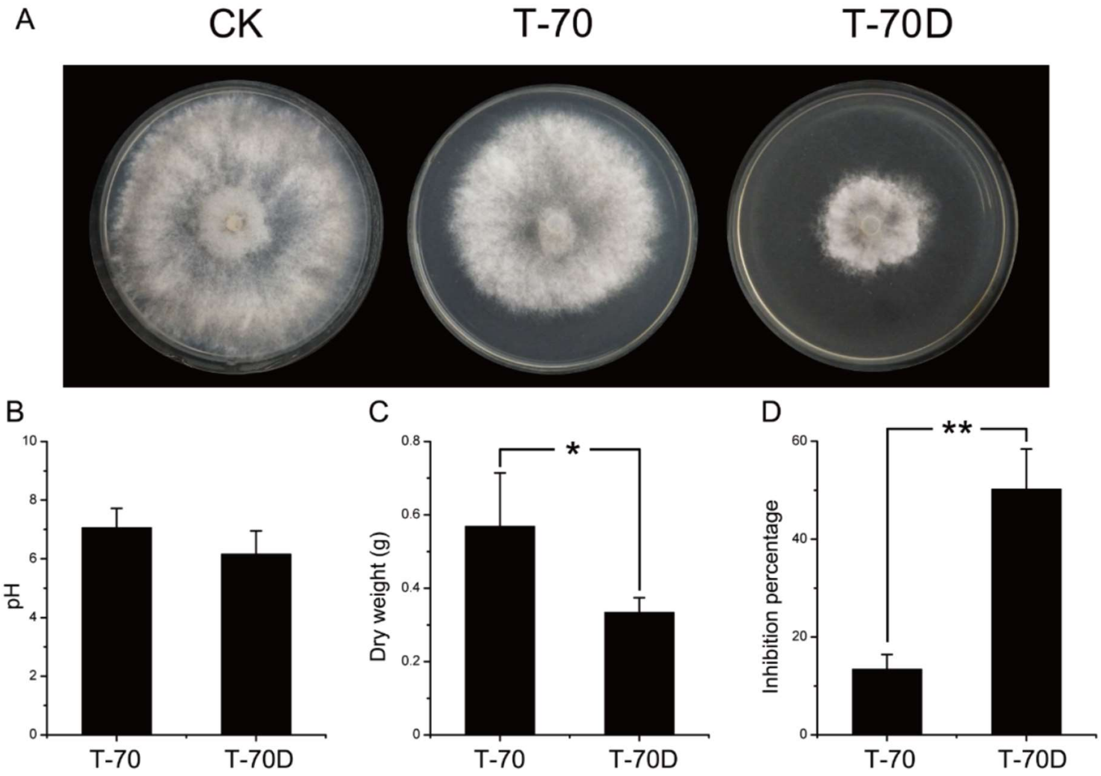

Figure S4. The antifungal ability assay of isolates T-70 and T-70D. (**A**) Mycelial growth of *Botrytis cinerea* on the PDA plate amended with 10% (*v*/*v*) cultural filtrate of isolate T-70, T-70D, or water (CK). (**B**) The pH value of the cultural filtrate for each isolate. (**C**) Mycelial dry weight of T-70 and T-70D after cultured in potato dextrose broth shake-incubated at 150 rpm on 25 °C for 7 days. (**D**) Inhibition percentage of *B. cinerea* growth rate on the PDA plate amended with10% (*v*/*v*) cultural filtrate of T-70 or T-70D. "*" and "**" indicate significant difference according to the Student t test at $p < 0.05$ and $p < 0.01$ ($n = 9$), respectively.

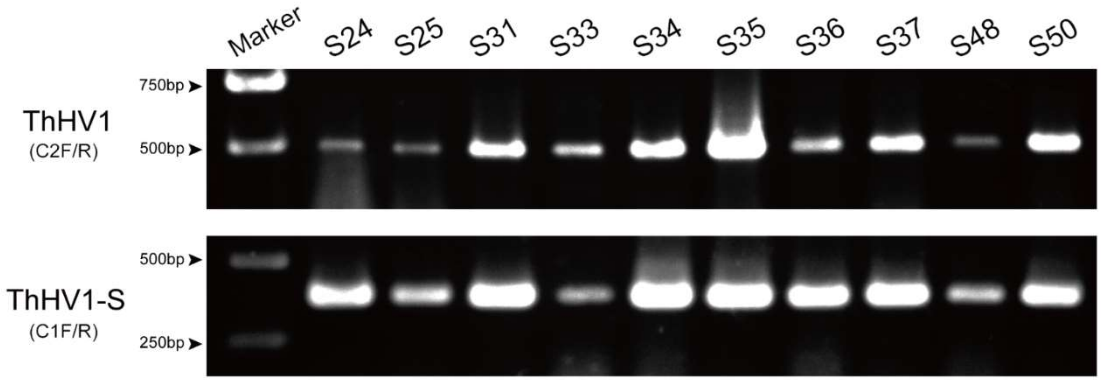

Figure S5. RT-PCR detection of presence of Trichoderma harzianum hypovirus 1 (ThHV1) and ThHV1-S in ten randomly selected single-conidium progeny isolates of T-70D with primer pairs C1F/C1R and C2F/C2R.

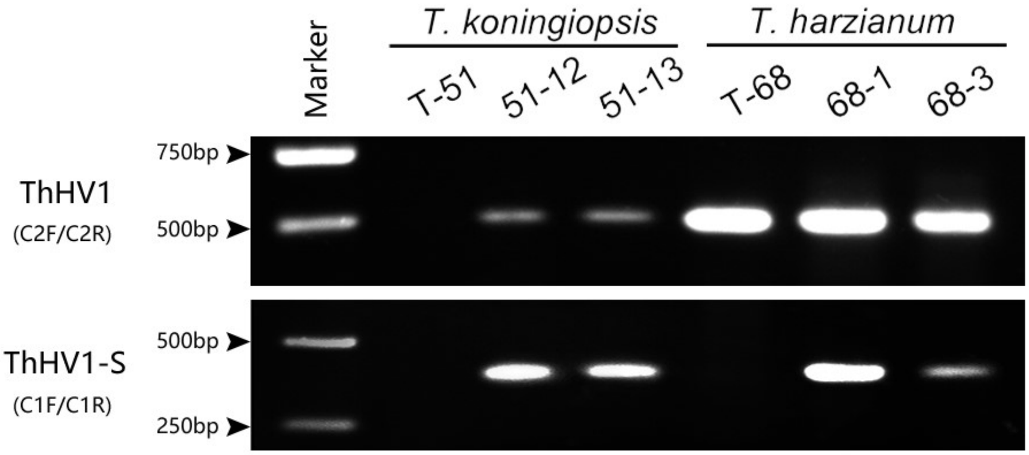

Figure S6. Detection of Trichoderma harzianum hypovirus 1 (ThHV1) and ThHV1-S in *Trichoderma* isolates T-68 and T-51 as well as their derivative strains using RT-PCR.

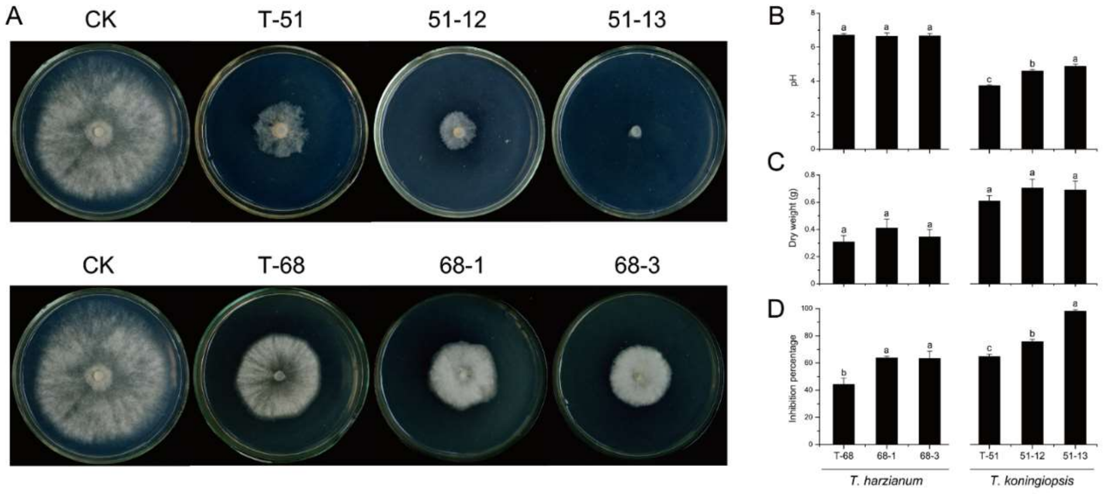

Figure S7. The antifungal ability assay of *Trichoderma* isolates T-68 and T-51 as well as their derivative strains. (**A**) Mycelial growth of *B. cinerea* on the PDA plate amended with 10% (*v*/*v*) cultural filtrate of each isolate/strain or water (CK). (**B**) The pH value of the cultural filtrate for each isolate/strain. (**C**) Mycelial dry weight of each isolate/strain after cultured in potato dextrose broth shake-incubated at 150 rpm on 25 °C for 7 days. (**D**). Inhibition percentage of *B. cinerea* growth rate on the PDA plate amended with 10% (*v*/*v*) cultural filtrate of each isolate/strain. Bars in graph **A**, **B** and **C** labeled with the same letters are not significantly different ($p > 0.05$) according to Least Significant Difference Test ($n = 9$).

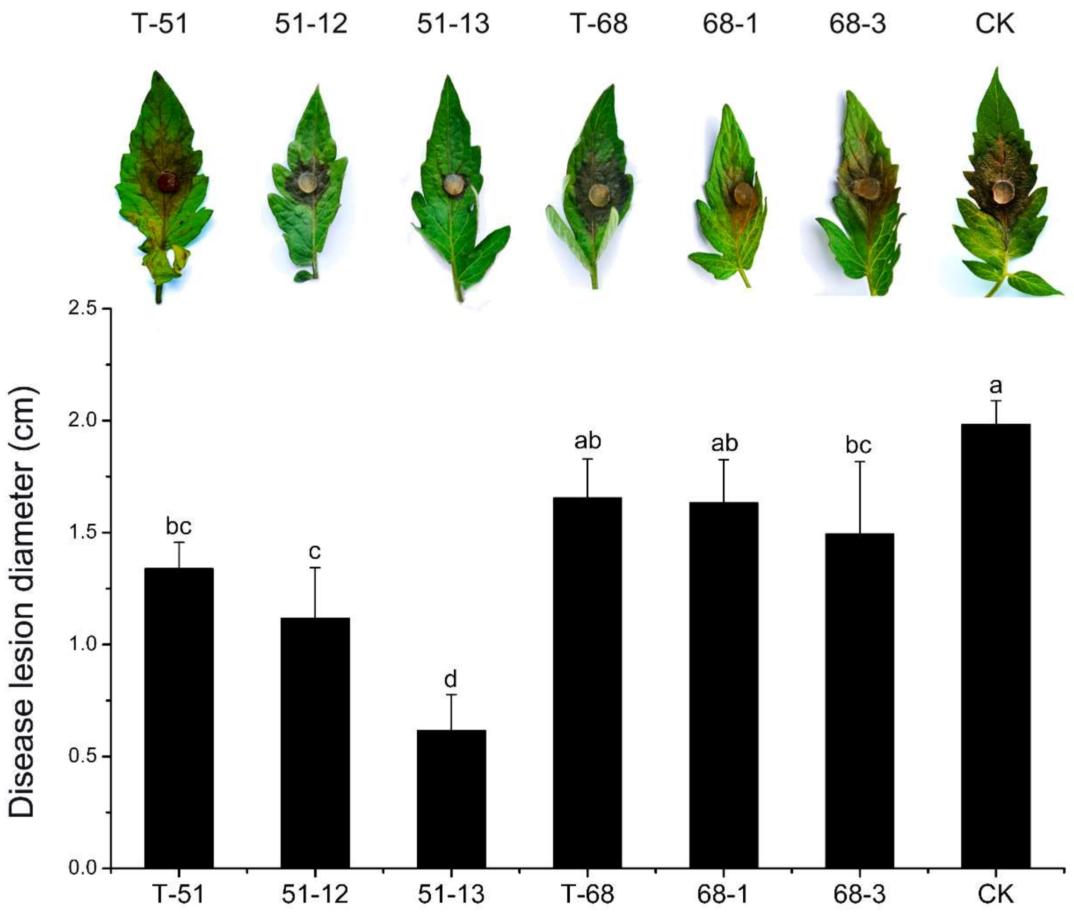

Figure S8. Antifungal ability of cultural filtrate of different *Trichoderma* isolates on tomato leaves against the infection of *B. cinerea*. Bars labeled with the same letters are not significantly different ($p <$ 0.05) according to Least Significant Difference Test ($n = 9$).

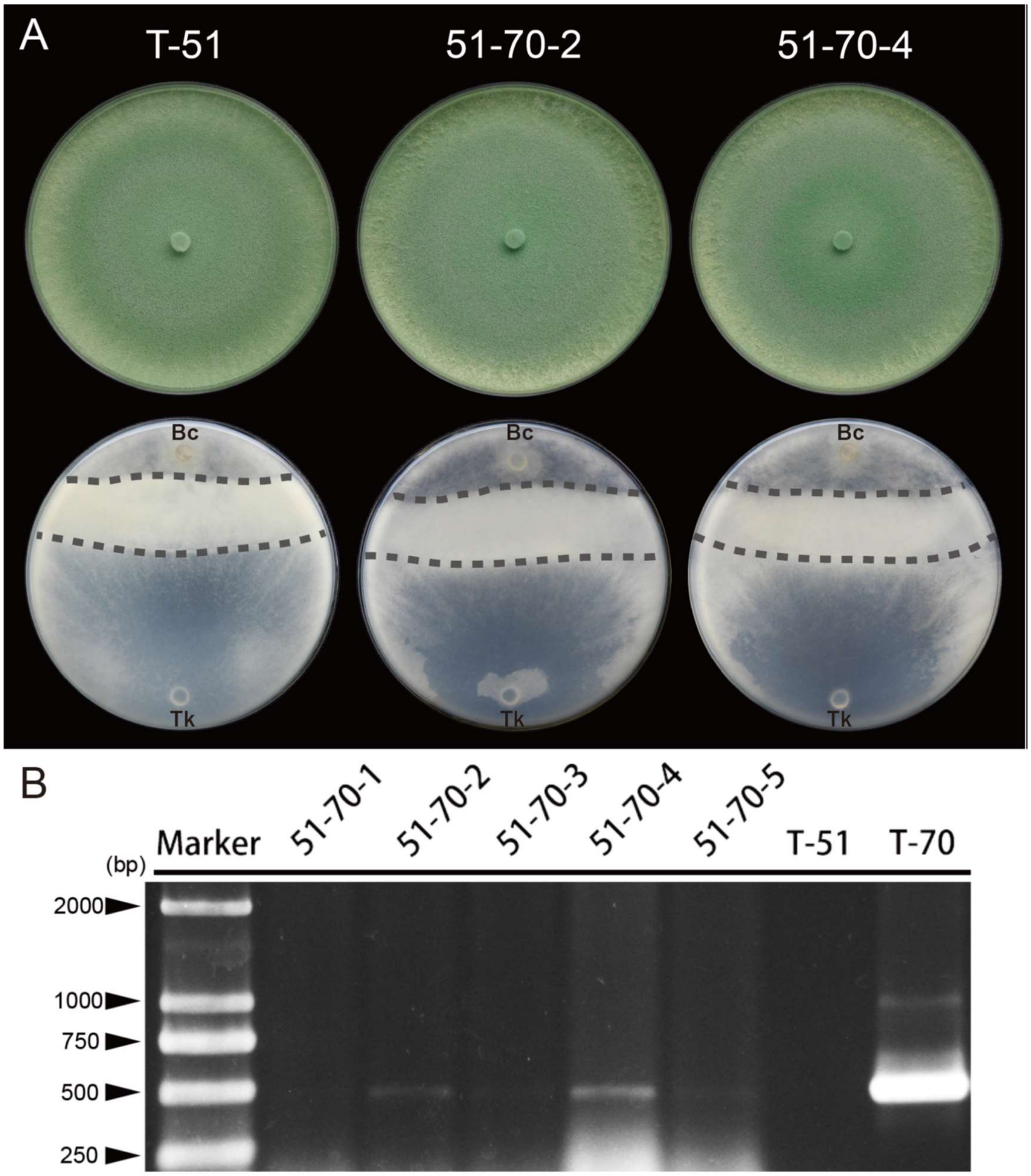

Figure S9. (**A**) Colony morphology and mycoparasitism ability of *Trichoderma koningiopsis* isolate T-51 and two derivative strains, 51-70-2 and 51-70-4, carrying Trichoderma harzianum hypovirus 1 (ThHV1) alone. The dashed lines indicate the regions of *B. cinerea* colonies colonized by *T. koningiopsis* in the three dual cultures. (**B**) RT-PCR detection the present of ThHV1 with primer pair C2F/C2R in isolates T-51 and T-70, and their derivative strains. Note that the 500-bp DNA band, indicating the presence of ThHV1, was detected in isolate T-70 and two derivative strains 51-70-2 and 51-70-4.
